# Supplementary material for: Bi-Allelic Novel Variants in CLIC5 Identified in a Cameroonian Multiplex Family with Non-Syndromic Hearing Impairment
Source: Genes (Basel). 2020 Oct 23;11(11):1249. doi: 10.3390/genes11111249 (PMC7690789; doi:10.3390/genes11111249)
Supplement: Supplementary file 1 [file genes-11-01249-s001.pdf]

SUPPLEMENTARY MATERIALS

Bi-allelic Novel Variants in *CLIC5* Identified in a Cameroonian Multiplex Family with Non-syndromic Hearing Impairment.

Edmond Wonkam-Tingang, Isabelle Schrauwen, Kevin K. Esoh, Thashi Bharadwaj, Liz M. Nouel-Saied, Anushree Acharya, Abdul Nasir, Samuel M. Adadey, Shaheen Mowla, Suzanne M Leal and Ambroise Wonkam

Table S1: Demographic and clinical characteristics of isolated NSHI cases screened for the identified *CLIC5* pathogenic variants. Mean age = 10.92 ± 4.84 (3 – 31) years.

**Categories**	**n/N**	**Frequency (%)**
**Age range (years)**		
<10	51/118	43.22
10 – 20	61/118	51.69
>20	6/118	5.08
**Gender**		
Male	63/118	53.39
Female	55/118	46.61
**Ethnic group**		
Bamileke (Semi-bantu)	49/118	41.53
Beti-fang (Bantu)	34/118	28.81
Bassa (Bantu)	11/118	9.32
Bamun (Semi-bantu)	9/118	7.63
Duala (Bantu)	7/118	5.93
Fulani (Sudanese)	4/118	3.39
Tikar (Semi-bantu)	3/118	2.54
Mbo (Bantu)	1/118	0.85
**Age of onset**		
Congenital/Prelingual (before 2 years old)	107/118	90.68
Perilingual (between 2 and 4 years)	9/118	7.63
Postlingual (after 4 years)	2/118	1.69
**Degree of hearing impairment**		
Moderate II	1/65	1.54
Severe I	4/65	6.15
Severe II	4/65	6.15
Profound I	14/65	21.54
Profound II	19/65	29.23
Profound III	12/65	18.46
Total	11/65	16.92
**Type of hearing impairment**		
Sensorineural	58/65	89.23
Mixed	7/65	10.77
Bilateral	65/65	100.00

Table S2: Description of pathogenic variants identified in *CLIC5*

	**c.224T>C [p.(L75P)]**	**c.63+1G>A**
Predicted effect	Missense	Splicing
Frequency in gnomAD	Absent	Absent
Frequency in UK10K	Absent	Absent
Frequency in GME	Absent	Absent
dbSNP rs number	Absent	Absent
GERP	5.73	4.72
PhyloP	9.29	4.49
PhastCons	1	1
SiPhy	15.30	12.06
SIFT	Damaging	NA
Polyphen2 HDIV	Probably damaging	NA
Polyphen2 HVAR	Probably damaging	NA
MutationAssessor	High	NA
LRT	Deleterious	NA
M-CAP	Damaging	NA
REVEL	Pathogenic	NA
MutPred	Pathogenic	NA
PROVEAN	Damaging	NA
MetaSVM	Damaging	NA
MetaLR	Tolerated	NA
MutationTaster	Disease causing	Disease causing
Eigen	Pathogenic	Benign
Eigen-PC	Pathogenic	Pathogenic
FATHMM-MKL	Damaging	Damaging
CADD	32	20.9
DANN	0.999	0.987
ACMG classification	Pathogenic	Pathogenic

NA, not applicable. RefSeq transcript used: NM_016929.5

Other variants that co-segregate with the hearing impairment within "Family 24"

Apart from *CLIC5*, only *CEP250* gene shows compound synonymous variants (i.e. NM_007186.5:c.1380T>C and NM_007186.5:c.1935C>T; Table S3) that co-segregate with hearing impairment, and was unlikely the cause of the disease. None of these synonymous variants is predicted to alter the splicing of the pre-mRNA. No homozygous variants segregate with the hearing impairment phenotype.

Table S3: Synonymous likely benign variants identified in *CEP250* gene

	**c.1380T>C [p.(S460S)]**	**c.1935C>T [p.(V645V)]**
Predicted effect	Synonymous	Synonymous
Frequency in gnomAD	0.0003	0.0005
Frequency in gnomAD_Afr	0.0009	0.0002
Frequency in GME	Absent	Absent
dbSNP rs number	rs142139756	rs370009858
ACMG classification	Likely benign	Likely benign

RefSeq used: NM_007186.5

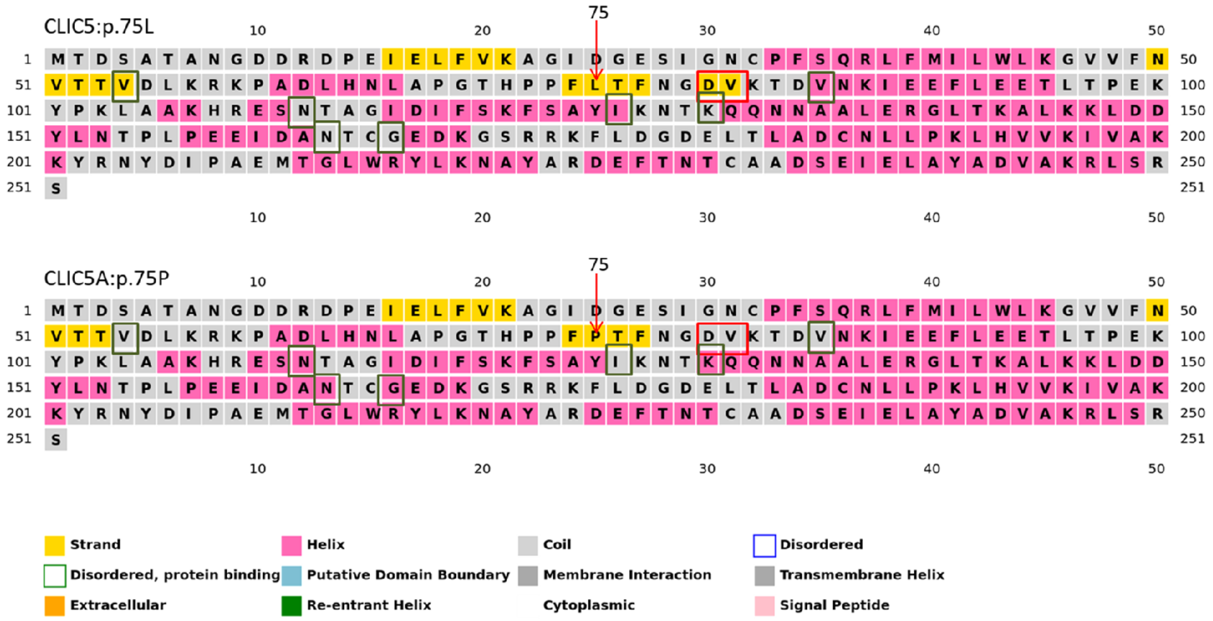

Figure S1: Secondary structure prediction of CLIC5 using the 251 amino acids isoform (NM_016929.5). Boxes indicate positions of difference between wild type (CLIC5A:p.75L) and mutant (CLIC5A:p.75P). Red boxes show loss of the fourth strand in the wild type while black boxes show changes in the lengths of strands and helices

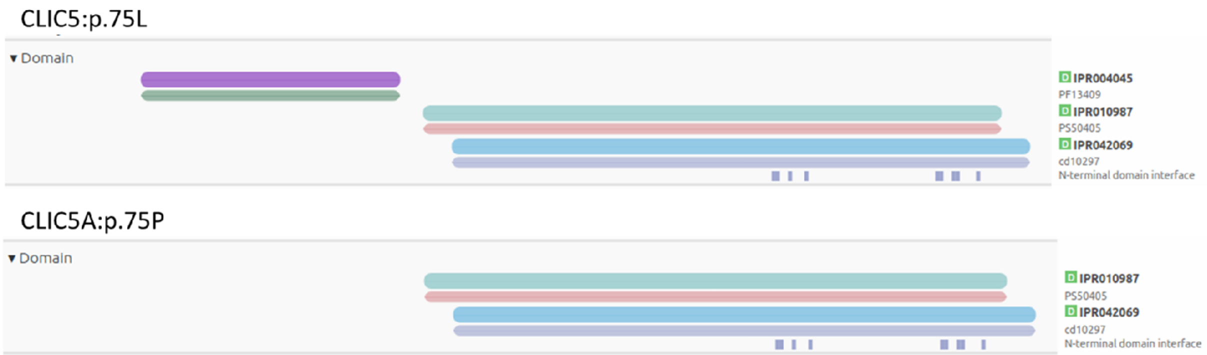

IPR004045: Glutathione S-transferase, N-terminal
IPR010987: Glutathione S-transferase, C-terminal-like
IPR042069: CLIC5, C-terminal GST domain
cd10297: C-terminal, alpha helical domain of Chloride Intracellular Channel 5

Figure S2: Domains of CLIC5A:p.75L (wild type) and CLIC5A:p.75P (mutant) predicted by InterPro, based on the 251 amino acids isoform (NM_016929.5). The GST N-terminal domain is lost in the mutant and its protein-binding activity is abolished.

CLIC5 physicochemical properties

Using ProtParam of the ExPASy Bioinformatics Resource Portal, CLIC5 wild type and mutant proteins were predicted to have similar physicochemical properties as expected including; pI (5.70), molecular weight (MW = 28.2kDa) and extinction coefficients. However, they differed on instability classification. While the wild type protein was classified as stable (stability index = 39.34), the mutant protein was classified as unstable (stability index = 40.11). This was consistent with the I-MutantSuite (I-Mutant3.0) result which predicted the variant to impose a "Large Decrease" in protein stability.

CLIC5 Protein structure determination and analysis

Given the predicted secondary structural changes, it is conceivable that there would be a three dimensional (3D) structural change of the protein due to the mutation. Considering that the structure of CLIC5A has been recently solved (PDB: 6Y2H), we used a single template-based approach, utilizing this structure as template to model the structures of the wild type and mutant (CLIC5:pL75P) proteins. 6Y2H is a 236 amino acid (CLIC5A residues 16 - 251) X-ray crystallographic monomeric structure resolved to 2.15 angstroms with zero (0) Ramachandran outliers.

As expected, the global model quality estimation (GMQE) for the wild type (0.93) and mutant (0.93) models were comparable to the experimentally-determined structure (0.96; on a scale of 0 – 1) (Table S4). The model quality was generally better for the wild type as compared to the mutant, while both models received Swiss-Model "thumbs up" for QMEANs indicating high quality. Following refinement, a considerable increase in the quality of the models was achieved as was apparent by zero Ramachandran outliers and poor rotamers, lower MolProbity and clash scores, and high Ramachandran favored and favored rotamer scores. The relatively lower Galaxy energy for the wild type model indicates a higher stability as compared to the mutant model, consistent with earlier findings.

Table S4: Model parameters before and after refinement showing improvement in model qualities

Parameter	Before Refinement		After Refinement	
	**CLIC5A:p.75L**	**CLIC5A:p.75P**	**CLIC5A:p.75L**	**CLIC5A:p.75P**
Galaxy energy	-	-	-6489.70	-6409.60
GMQE (0-1)	0.93	0.93	-	-
QMEAN (Goal: 0)	-1.45 (abs 1.45)	-1.53 (abs 1.53)	-	-
MolProbity (smaller is better)	0.85 (100th percentile)	1.02 (100th percentile)	0.50 (100th percentile)	0.75 (100th percentile)
Clash score (Goal: 0)	1.07 (99th percentile)	1.61 (99th percentile)	0 (100th percentile)	0.81 (99th percentile)
Ramachandran favored (Goal: >98%)	97.84%	97.41%	99.57%	98.28%
Poor rotamers (<0.3%)	0.50%	0.50%	0.00%	0.00%
Favored rotamers (>98%)	91.54%	92.04%	100%	99.50%
Ramachandran outliers (Goal: <0.05%)	0.43% (1)	0.86% (2)	0.00%	0.00%
Rama distribution Z-score (Goal: <2)	1.25 ± 0.53	1.22 ± 0.53	0.23 ± 0.50	0.31 ± 0.51
C-beta deviations (Goal: 0)	4	3	1	0
Bad bonds (Goal: 0%)	0.16%	0.16%	0.00%	0.00%
Bad angles (Goal: <0.1%)	0.90%	0.97%	0.47%	0.35%

GMQE, global model quality estimation; QMEAN, qualitative model energy analysis
